# Allopurinol and oxypurinol promote osteoblast differentiation and increase bone formation

**DOI:** 10.1016/j.yexcr.2016.03.004

**Published:** 2016-03-15

**Authors:** Isabel R. Orriss, Timothy R. Arnett, Jacob George, Miles D. Witham

**Affiliations:** a Department of Comparative Biomedical Sciences, Royal Veterinary College, London NW1 0TU, UK; b Department of Cell & Developmental Biology, University College London, London, UK; c Medical Research Institute, University of Dundee, Dundee, UK

**Keywords:** Bone formation, Allopurinol, Oxypurinol, Osteoblast differentiation

## Abstract

Allopurinol and its active metabolite, oxypurinol are widely used in the treatment of gout and hyperuricemia. They inhibit xanthine oxidase (XO) an enzyme in the purine degradation pathway that converts xanthine to uric acid. This investigation examined the effect of allopurinol and oxypurinol on bone formation, cell number and viability, gene expression and enzyme activity in differentiating and mature, bone-forming osteoblasts. Although mRNA expression remained relatively constant, XO activity decreased over time with mature osteoblasts displaying reduced levels of uric acid (20% decrease). Treatment with allopurinol and oxypurinol (0.1–1 µM) reduced XO activity by up to 30%. At these concentrations, allopurinol and oxypurinol increased bone formation by osteoblasts ~4-fold and ~3-fold, respectively. Cell number and viability were unaffected. Both drugs increased tissue non-specific alkaline phosphatase (TNAP) activity up to 65%. Osteocalcin and TNAP mRNA expression was increased, 5-fold and 2-fold, respectively. Expression of NPP1, the enzyme responsible for generating the mineralisation inhibitor, pyrophosphate, was decreased 5-fold. Col1α1 mRNA expression and soluble collagen levels were unchanged. Osteoclast formation and resorptive activity were not affected by treatment with allopurinol or oxypurinol. Our data suggest that inhibition of XO activity promotes osteoblast differentiation, leading to increased bone formation *in vitro*.

## Highlights

• Allopurinol and oxypurinol are inhibitors of xanthine oxidase.
• Treatment with allopurinol and oxypurinol increases bone formation by osteoblasts *in vitro.*
• The expression and activity of alkaline phosphatase is stimulated by allopurinol and oxypurinol.
• Osteoclast formation and activity is unaffected by either drug.
• Inhibition of xanthine oxidase promotes osteoblast differentiation, leading to increased bone formation.

## 1 Introduction

Most of the current treatments for postmenopausal osteoporosis (*e.g.* bisphosphonates, Denosumab) act by inhibiting osteoclast activity and reducing bone resorption, thereby increasing bone mineral density (BMD). In contrast, the only bone anabolic agent currently marketed for treating osteoporosis is the human parathyroid hormone (PTH) analogue teriparatide [1], [2]. Since use of PTH is not suitable for all patients [3], additional therapeutic agents which promote bone formation are required.

Allopurinol (1,5-dihydro-4*H*-pyrazole[3,4-*d*]pyrimidin-4-one) and its active metabolite oxypurinol are widely used clinically in the treatment of gout, the most common form of inflammatory arthritis, and hyperuricemia [4], [5]. Both agents are purine analogues and act as non-competitive inhibitors of xanthine oxidase (XO), an enzyme in the purine degradation pathway. Febuxostat, which is structurally unrelated to allopurinol, is a non-purine selective XO inhibitor also used to treat gout [6].

Physiologically, XO is involved in many biochemical reactions but its key action is to catalyse the breakdown of hypoxanthine to xanthine and xanthine to uric acid [7]. Inhibition of XO activity reduces the uric acid concentration in the plasma and therefore prevents the development and progression of gout and related conditions [4]. XO expression is widely distributed throughout the body with expression in the liver, gut, lung, kidney, heart and brain [7]. Expression of XO has also been reported in osteoblasts and osteoclasts [8]. Inherited deficiency of XO activity leads to xanthinuria and multiple organ failure characterised by low levels of uric acid and an accumulation of xanthine in tissues [9].

The breakdown of hypoxanthine and xanthine by XO is an oxygen-dependent reaction that also results in the production of the reactive oxygen species (ROS) superoxide (O_2_^−^) and hydrogen peroxide. XO-derived superoxide can cause oxidative injury to proteins, lipids and DNA, so in preventing its production allopurinol and oxypurinol can act as powerful antioxidants [7]. Previous work seems to suggest that XO activity mainly exerts negative effects on bone. In osteoblast-like cells and bone marrow stromal cells, XO increases oxidative stress leading to reduced cell viability, an inhibition of differentiation and decreased expression of osteogenic markers [10], [11], [12]. Furthermore, osteoblast XO activity is enhanced by inflammatory cytokines including TNFα and IL-1β [8]. XO-derived superoxide has also been shown to stimulate the expression of receptor activator of nuclear factor _Κ_B ligand (RANKL) in osteoblast-like cells [13]. In osteoclasts, ROS that can be generated by XO have been shown to increase formation and bone resorption [14], [15].

Despite gout being a condition that primarily affects the musculoskeletal system, the effects of allopurinol and oxypurinol on bone remain poorly investigated. Whilst there are no studies describing the direct effects of these drugs on bone cell function, allopurinol has been shown to inhibit the increase in bone resorption caused by TNFα and IL-1β [8]. More recently, a combination of allopurinol and another antioxidant, *N-*acetylcysteine, was found to inhibit bone growth in an immobilisation-manipulation model of heterotopic ossification [16].

The aim of this study was to examine the direct effects of allopurinol and oxypurinol on osteoblast and osteoclast survival, differentiation and function, using established *in vitro* methods.

## 2 Materials and methods

### 2.1 Reagents

All tissue culture and molecular biology reagents were purchased from Life Technologies (Paisley, UK) unless stated otherwise. Chemical reagents were purchased from Sigma Aldrich (Poole, UK).

### 2.2 Osteoblast cell culture

Primary rat osteoblast cells were obtained from 2-day-old neonatal Sprague-Dawley rats euthanised by cervical dislocation, as described previously [17], [18]. All animal experiments were approved by the University College London Animal Users Committee and the Royal Veterinary College ethics and welfare committee; all animals were maintained in accordance with the UK Home Office guidelines for the care and use of laboratory animals.

Following isolation, cells were resuspended in Dulbecco's Modified Essential Medium, supplemented with 10% foetal calf serum (FCS), 2 mM l-glutamine, 100 U/ml penicillin, 100 µg/ml streptomycin and 0.25 µg/ml amphotericin (complete mixture abbreviated to DMEM). Cells were cultured for 2–4 days in a humidified atmosphere of 5% CO_2_–95% air at 37 °C in 75 cm^2^ flasks until confluent. Upon confluence, cells were sub-cultured into 24-well trays in DMEM supplemented with 2 mM β-glycerophosphate, 50 μg/ml ascorbic acid and 10 nM dexamethasone (supplemented DMEM), with half medium changes every 3 days. Osteoblasts were cultured in the presence of allopurinol and oxypurinol (1 nM–10 µM) to determine the effect on cell proliferation, differentiation, function and gene expression. For the bone formation experiments, cells were also treated with febuxostat and, as a positive control of an anabolic agent, BMP2 (0.1 µM). Unless stated, experiments were carried out at 2 time points during the osteoblast culture; day 7, which represents differentiating osteoblasts, and day 14 (mature, bone forming osteoblasts). All experiments were carefully pH-controlled because bone mineralisation is extremely sensitive to inhibition by acidosis [19]. Bone nodule formation by osteoblasts cultured in 24-well plates was measured by image analysis as described previously [17], [18], [20].

### 2.3 Osteoclast cell culture

The long bones were dissected from 6 week-old mice, cut across the epiphyses and the marrow was flushed out with PBS. The resulting suspension was centrifuged at 1500 rpm and resuspended in αMEM supplemented with 100 nM prostaglandin E_2_ (PGE_2_) and 50 ng/ml macrophage colony stimulating factor (M-CSF). The cell suspension was cultured for 24 h in a 75 cm^2^ flask in 5% CO_2_/95% atmospheric air to allow attachment of stromal cells and other rapidly adherent cells. The non-adherent cell suspension was removed, centrifuged and resuspended in αMEM supplemented with 100 nM PGE_2_, 200 ng/ml M-CSF and 3 ng/ml RANKL (R&D Systems Europe Ltd, Abingdon, UK). Cells were plated onto 5 mm diameter ivory discs (10^6^ cells/disc) in 96-multiwells. After 24 h, discs containing adherent osteoclast precursors were transferred to 6 well trays (4 discs/well in 4 ml medium) for a further 6 days at 37 °C in 5% CO_2_/95% atmospheric air. Culture medium was acidified to pH ~7.0 by the addition 10 meq/l H^+^(as HCL) on day 7 to activate osteoclasts to resorb dentine [21]. Culture medium pH, pCO_2_ and PO_2_ were monitored throughout using a blood gas analyser (ABL 705, Radiometer, Copenhagen, Denmark). Allopurinol or oxypurinol (1 nM–10 µM) were added for the duration of the culture.

Osteoclasts were fixed in 2% glutaraldehyde and stained to demonstrate tartrate-resistant acid phosphatase (TRAP). Osteoclasts were defined as TRAP-positive cells with 2 or more nuclei and/or clear evidence of resorption pit formation. Osteoclast number and the area resorbed on each disc were assessed ‘blind’ by transmitted light microscopy and reflective light microscopy and dot-counting morphometry, respectively [21].

### 2.4 Measurement of xanthine oxidase (XO) activity

Osteoblasts were cultured with 0.1–1 µM allopurinol and oxypurinol for 7 or 14 days. The XO activity of cell lysates was determined colorimetrically using a commercially available kit (XO assay kit, Abcam, Cambridge UK). Total protein in cell lysates was determined using the Bradford assay (Sigma Aldrich, Poole, UK).

### 2.5 Cell number and viability assay

Osteoblast cell number was measured after 7 and 14 days of treatment with allopurinol and oxypurinol (1 nM–10 µM) using the CytoTox 96® non-radioactive cytotoxicity assay (Promega UK, Southampton UK). This assay quantifies cellular lactate dehydrogenase (LDH), a stable cytosolic enzyme that is released on cell lysis. LDH oxidises lactate into pyruvate, generating NADH, which is then used to convert a tetrazolium salt into a red formazan product in proportion to the number of lysed cells.

Cell supernatants were collected to determine medium LDH levels (cell viability). To establish total cellular LDH levels, cells were lysed with 1% Triton X-100 in water (lysis buffer, 15 µl/ml of medium) for 1 h. The LDH content of the supernatants and cell lysates were measured colorimetrically (490 nm) as per manufacturer's instructions. A standard curve for determination of cell numbers was constructed using cells seeded at 10^2^–10^6^/well. By expressing medium LDH as a percentage of the total cellular LDH cell viability could be also calculated.

### 2.6 Measurement of extracellular ATP

Prior to measurement of ATP levels, culture medium was removed, cell layers washed and cells incubated with serum-free DMEM (1 ml/well) for 1 h. Extracellular ATP release was measured luminometrically using the *luciferin-luciferase* assay as previously reported [22]. Cell number and viability were determined as described above.

### 2.7 Determination of alkaline phosphatase (TNAP) activity

The TNAP activity of cell lysates was determined colorimetrically using a commercially available kit (SensoLyte® pNPP TNAP assay kit, Anaspec, Fremont, CA); this assay uses p-nitrophenyl phosphate as a substrate, which in the presence of TNAP, is converted to the yellow chromogen p-nitrophenyl. Osteoblast TNAP activity was measured after 7 and 14 days of culture. Cell layers were washed and cells harvested using a scraper (n=6) followed by sonication at 4 °C and centrifugation at 500×*g*. The supernatant was collected and stored at 4 °C until assaying at pH 9.8. Total protein in cell lysates was determined using the Bradford assay.

### 2.8 Measurement of collagen production

Soluble collagen production was determined in osteoblasts after 7 and 14 days of culture with 10 nM–1 µM allopurinol or oxypurinol; total protein concentration in lysates was determined using the Bradford assay. Osteoblasts were transferred to medium containing 5% FCS, 2 mM β-glycerophosphate, 50 μg/ml ascorbic acid, 10 nM dexamethasone and the lysyl oxidase inhibitor β-aminopropionitrile (50 μg/ml) for the final 24 h of culture. Medium without cells was used as a blank. The concentration of collagen accumulated in the tissue culture medium was assayed using a Sirius red dye-based kit (Sircol soluble collagen assay, Biocolor Ltd., Newtownabbey, UK) according to the manufacturer's instructions.

### 2.9 Total RNA extraction and Dnase treatment

Osteoblasts were cultured in 6-well trays for 7 or 14 days with 0.1 µM allopurinol or oxypurinol; total RNA was extracted from 3 wells using TRIZOL^®^ reagent (Invitrogen, Paisley, UK) according to the manufacturer's instructions. Extracted RNA was treated with RNase-free DNase I (35 U/ml) for 30 min at 37 °C. The reaction was terminated by heat inactivation at 65 °C for 10 min. Total RNA was quantified spectrophotometrically by measuring absorbance at 260 nM. RNA was stored at −80 °C until amplification by qPCR.

### 2.10 Quantitative real time polymerase chain reaction (qPCR)

Osteoblast RNA (50 ng) was transcribed and amplified using the iScript one-step qRT-PCR kit with SYBR green (Biorad Laboratories Ltd., Hemel Hempstead, UK), which allows cDNA synthesis and PCR amplification to be carried out sequentially. qRT-PCR (chromo4, Biorad Laboratories Ltd., Hemel Hempstead, UK) was performed according to manufacturer's instructions with initial cDNA synthesis (50 °C for 10 min) and reverse transcriptase inactivation (95 °C for 5 min) followed by 40 cycles of denaturation (95 °C for 10 s) and detection (60 °C for 30 s). Gene expression was investigated in cells cultured for 7 and 14 days. Data was analysed using the Pfaffl method [23] and is shown as changes in the level of gene expression relative to untreated cells. All reactions were carried out in triplicate using RNAs derived from 4 different osteoblast cultures. Primer sequences: *β-actin,* S: *gcc ttc ctt cct ggg tat gg*/ AS: *tcc gat tca act cat act gc*; *COL1α1*, S: *ggg aca cag agg ttt cag tgg*/ AS: *agc tcc att ttc acc agg act g*; *TNAP*, S: *aaa cct aga cac aag cac tc*/ AS: *tcc gat tca act cat act gc*; *XO*, S: *aca cca tga aaa ccc aga gc*/ AS: *tcc acc cat cct ctt cac tc*; *Ocn,* S: *gca gac acc atg agg acc ct*/ AS: *gca gct tgt gcc gtc cat ac*; *Npp1,* S: *aga cca cac ttt tac act ctg*/ AS: *gat gac ctc act gct tac tg*

### 2.11 Statistics

Statistical comparisons were made using one-way analysis of variance (ANOVA) with a post-hoc Bonferroni correction for multiple comparisons. Calculations were performed using In Stat 3 (GraphPad, San Diego, CA). All data are presented as means±SEM for between 6 and 12 replicates. Results are representative of experiments performed at least three times using cells isolated from different animals.

## 3 Results

### 3.1 Allopurinol and oxypurinol inhibit XO activity in osteoblasts

**Fig. 1:**
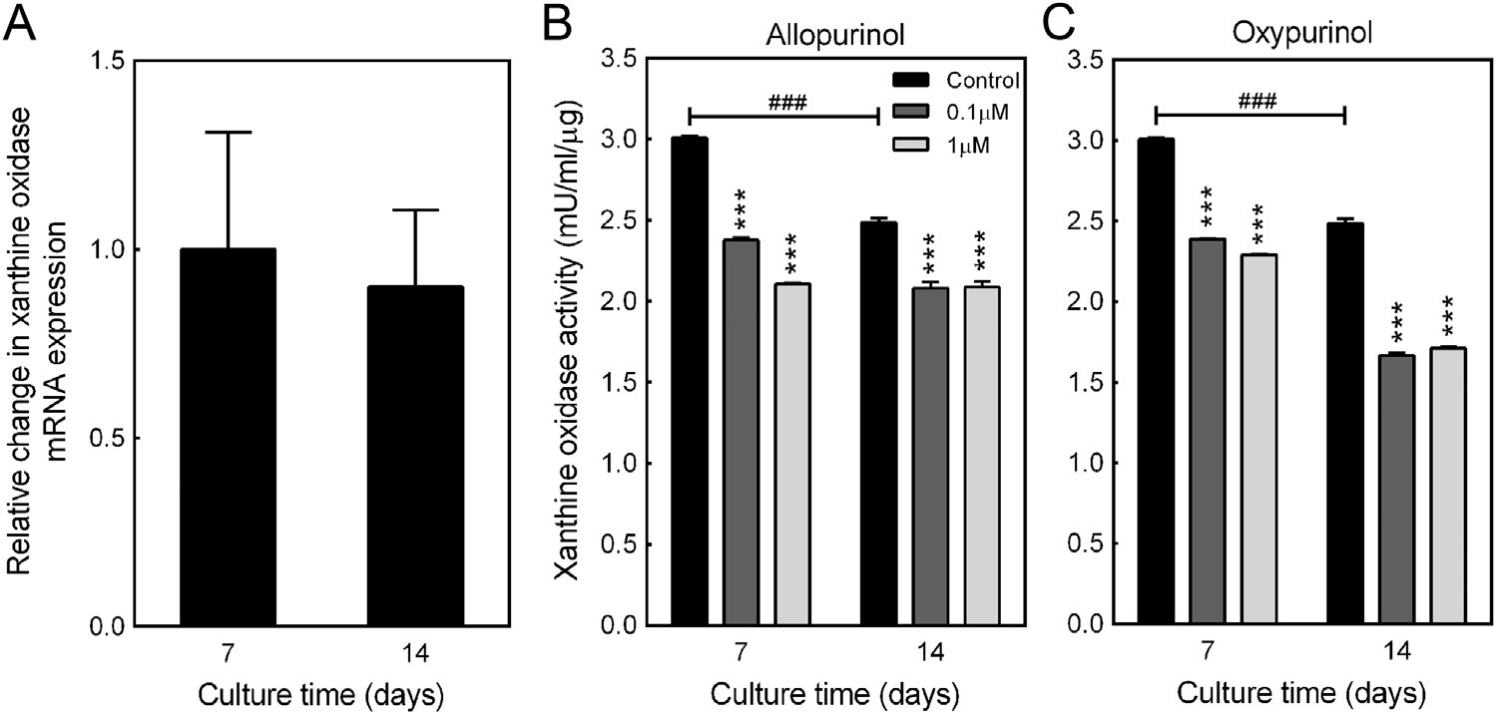
Allopurinol and oxypurinol inhibit osteoblast XO activity. (A) The level of XO mRNA expression is the same in differentiating cells (day 7 of culture) and mature, bone-forming osteoblasts (day 14). (B) XO activity was 20% lower in mature osteoblasts compared to differentiating cells. Allopurinol (≥0.1 µM) inhibits XO activity by 30% and 20% at day 7 and day 14, respectively. (C) Oxypurinol (≥0.1 µM) reduced XO activity by up to 32%. Values are means±SEM (n=6), ***/###=p<0.001, **=p<0.01, *=p<0.05.

Previous work has reported XO expression by osteoblasts [8]. In this investigation, XO mRNA expression and activity was measured in differentiating (day 7) and mature, bone forming osteoblasts (day 14). qPCR analysis of XO expression showed that mRNA levels were unaffected by osteoblast differentiation (Fig. 1A). However, enzyme activity was 20% lower in mature osteoblasts compared to differentiating cells (Fig. 1B and C). Allopurinol (≥0.1 μM) inhibits XO activity by 30% and 20% at day 7 and 14, respectively (Fig. 1B). Oxypurinol reduced XO activity by up to 32% (Fig. 1C).

### 3.2 Allopurinol and oxypurinol increase bone formation by osteoblasts

**Fig. 2:**
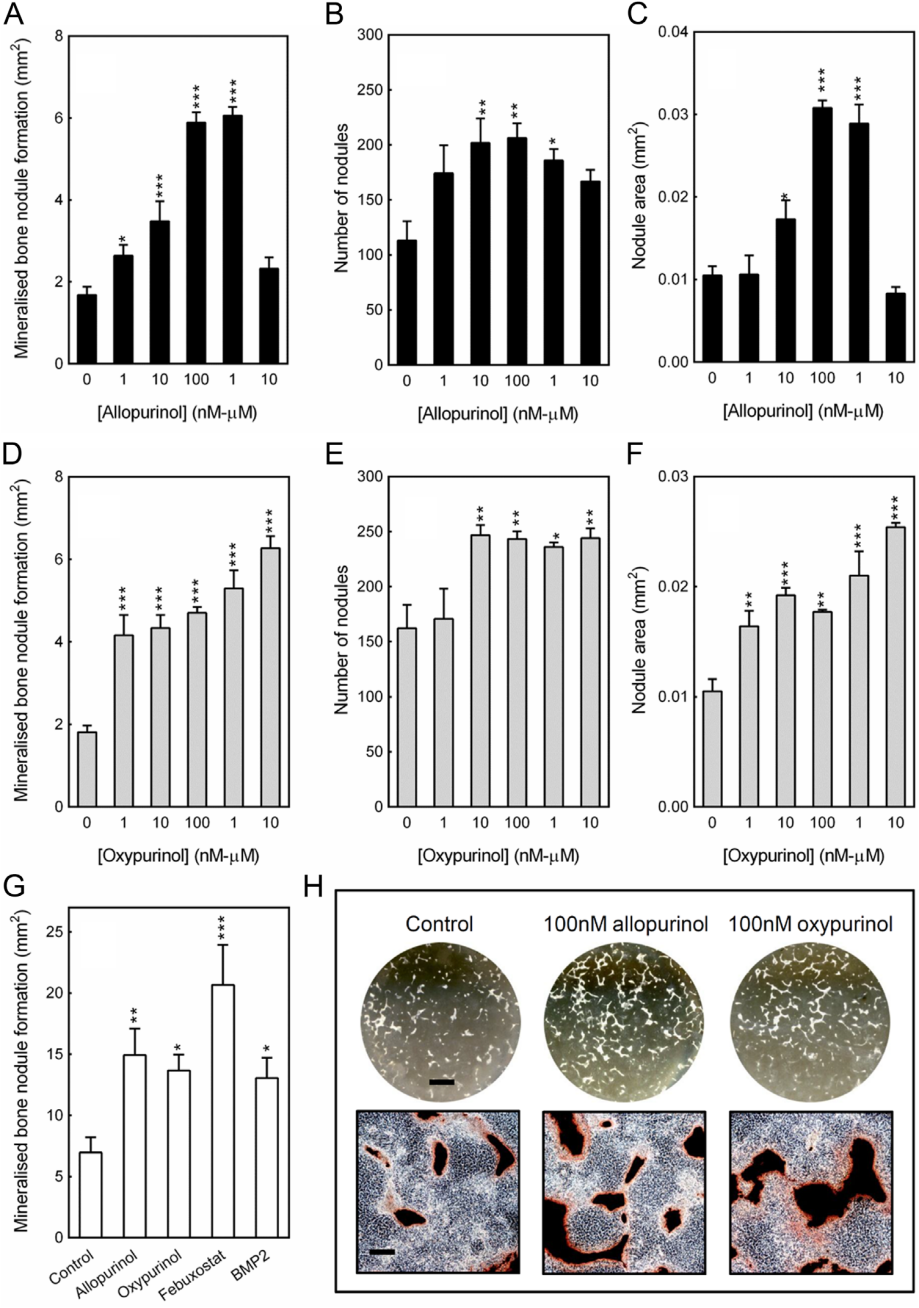
Allopurinol and oxypurinol increase bone formation by osteoblasts. (A) Allopurinol (≥1 nM) stimulates bone formation by up to 4-fold. No effect was seen with 10 µM allopurinol. The (B) number and (C) size of the mineralised nodules were also increased by allopurinol treatment (≥10 nM). (D) Oxypurinol (≥1 nM) increased bone formation by up to 3-fold; the (E) number and (F) size of the mineralised nodules were also increased. (G) Allopurinol, oxypurinol and BMP2 promoted bone formation to a similar extent (~2-fold). Febuxostat induced the largest increase in bone formation (3-fold). Values are means±SEM (n=6), ***=p<0.001, **=p<0.01, *=p<0.05. (H) Representative whole well scans (unstained) and phase contrast microscopy images (alizarin red stained) show the increased bone formation seen with allopurinol and oxypurinol. Scale bars: whole well=0.5 cm, phase contrast images=500 µm.

Rat calvarial osteoblasts were cultured for 14 days in the presence of 1 nM–10μM allopurinol or oxypurinol. Allopurinol (≥1 nM) dose-dependently increased bone formation up to 4-fold; this was due to an increase in the total number and size of the mineralised nodules. The peak stimulatory effects were seen at 0.1 µM and 1 µM; concentrations ≥10 μM had no effect (Fig. 2A–C, H). Treatment with oxypurinol also increased bone formation and mineralised nodule number and size: effects were evident from 1 nM with the maximal stimulation seen at 1–10 µM (3-fold increase) (Fig. 2D–F, H). For a comparative study of potency, allopurinol and oxypurinol were also cultured alongside BMP2 and febuxostat (0.1 µM). The stimulatory effects of allopurinol and oxypurinol were similar in magnitude to BMP2 (~2-fold), whilst febuxostat appeared most potent increasing bone formation 3-fold (Fig. 2G).

### 3.3 Allopurinol and oxypurinol do not affect osteoblast number, viability or ATP release

**Fig. 3:**
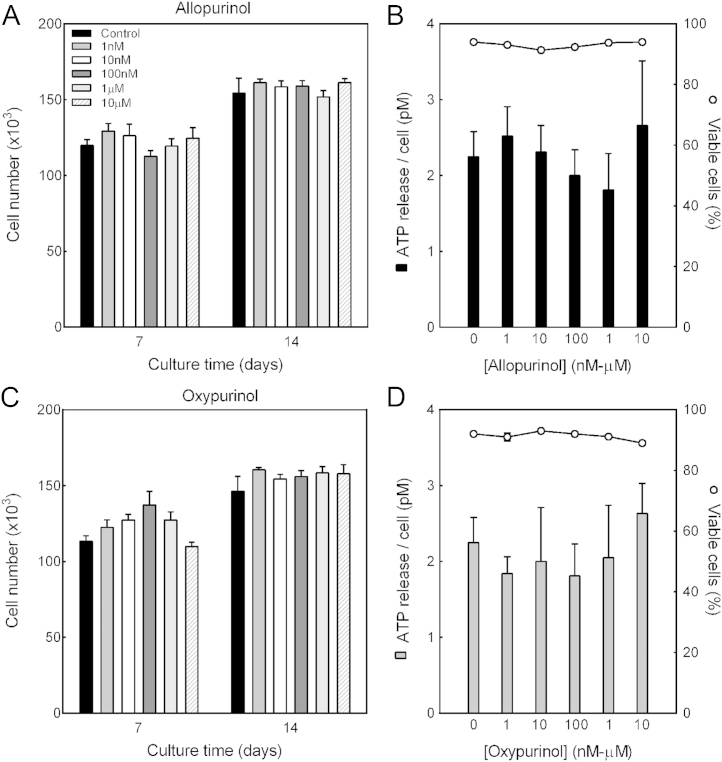
No effect of allopurinol or oxypurinol on cell number, viability or ATP release. Cell number was measured after 7 or 14 days of treatment with 1 nM–10 µM allopurinol or oxypurinol; ATP release and viability were assessed in mature osteoblasts. At all concentrations tested, (A-B) allopurinol and (C-D) oxypurinol had no effect on cell number, controlled ATP release (solid bars, primary y axis) or viability (lines, secondary y axis). Values are means±SEM (n=6–12).

To ensure prolonged exposure to allopurinol or oxypurinol (1 nM–10 μM) was not toxic to osteoblasts, cell number was measured after 7 and 14 days of treatment. At all concentrations tested allopurinol and oxypurinol did not influence cell number in differentiating (day 7) or mature bone-forming (day 14) osteoblasts (Fig. 3A and C).

Xanthine is formed by the breakdown of extracellular ATP or adenosine. Culture with allopurinol and oxypurinol had no effect on controlled ATP release from mature bone-forming osteoblasts; cell viability was also unaffected (Fig. 3B and D).

### 3.4 Allopurinol and oxypurinol stimulate osteoblast TNAP activity

**Fig. 4:**
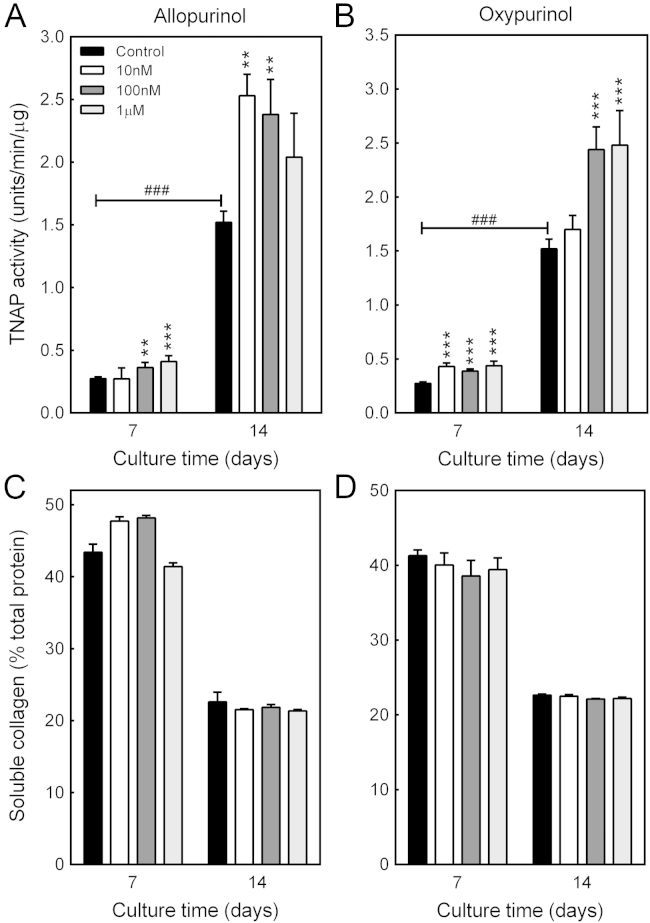
Increased TNAP activity in osteoblasts treated with allopurinol or oxypurinol. TNAP activity was measured in differentiating (day 7) and mature (day 14) osteoblasts treated with 10 nM–0.1 µM allopurinol or oxypurinol. Basal TNAP activity was ~6-fold higher in mature, bone-forming osteoblasts. (A) Allopurinol (≥10 nM) increased TNAP activity by up to 50% and 65% at day 7 and day 14, respectively. (B) Oxypurinol (≥10 nM) stimulated TNAP activity by ≤60% in differentiating cells and mature osteoblasts. (C) Allopurinol and (D) oxypurinol have no effect on soluble collagen levels. Values are means±SEM (n=6), ***/###=p<0.001, **=p<0.01.

TNAP activity was measured in differentiating and mature bone-forming osteoblasts treated with 10 nM–1 µM allopurinol or oxypurinol. Basal TNAP activity was 6-fold higher in mature osteoblasts compared to differentiating cells. Allopurinol (≥10 nM) stimulated TNAP activity by up to 50% and 65% in differentiating and mature cells, respectively (Fig. 4A). Oxypurinol (≥10 nM) increased osteoblast TNAP activity by up to 65% (Fig. 4B).

### 3.5 No effect of allopurinol and oxypurinol on soluble collagen

Soluble collagen levels were measured in cultures of osteoblasts treated with allopurinol or oxypurinol for 7 or 14 days. In both differentiating and mature osteoblasts there was no effect of either drug on collagen production (Fig. 4C and D).

### 3.6 The expression of key osteoblast genes is influenced by allopurinol and oxypurinol

**Fig. 5:**
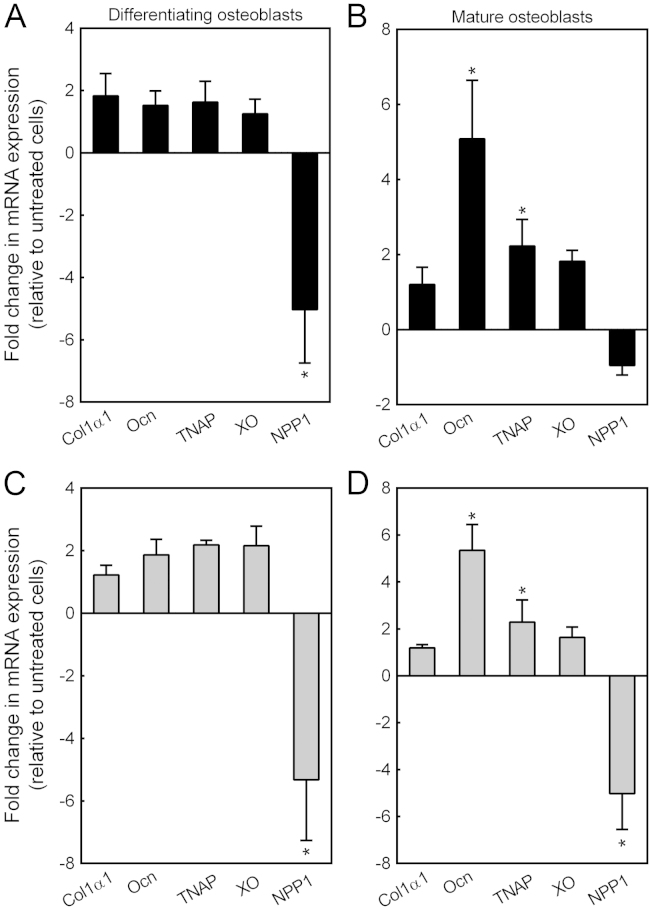
Allopurinol and oxypurinol influence the expression of key osteoblast genes. The effect of allopurinol and oxypurinol (0.1 μM) on the expression of collagen type I (Col1α1), osteocalcin (Ocn), TNAP, XO and ecto-nucleotide pyrophosphatase/phosphodiesterase 1 (NPP1) was in investigated in differentiating and mature osteoblasts. (A) Allopurinol decreased Npp1 expression ~5-fold in differentiating osteoblasts. (B) In mature osteoblasts, allopurinol increased TNAP and Ocn expression 2-fold and 5-fold, respectively. (C and D) Oxypurinol reduced Npp1 expression 5-fold in differentiating and mature osteoblasts. Levels of TNAP and Ocn expression were increased 2-fold and 5-fold, respectively in mature osteoblasts. Values are means±SEM (n=4), *=p<0.05.

The effect of allopurinol and oxypurinol (0.1 μM) on the expression of collagen type I (Col1α1), osteocalcin (Ocn), TNAP, XO and ecto-nucleotide pyrophosphatase/phosphodiesterase 1 (NPP1) was in investigated in differentiating (day 7) and mature, bone-forming osteoblasts (day 14). Allopurinol decreased NPP1 expression ~5-fold in differentiating osteoblasts; expression of *Col1α1, TNAP, Ocn* and *XO* was unaffected (Fig. 5A). In mature osteoblasts, allopurinol increased *TNAP* and *Ocn* expression 2-fold and 5-fold, respectively (Fig. 5B). Oxypurinol reduced *Npp1* expression 5-fold in differentiating and mature osteoblasts. Levels of *TNAP* and *Ocn* expression were increased 2-fold and 5-fold, respectively in mature osteoblasts. *Col1α1* and *XO* mRNA expression was unaffected by treatment with oxypurinol (Fig. 5C and D).

### 3.7 Osteoclast formation and activity is unaffected by treatment with allopurinol and oxypurinol

**Fig. 6:**
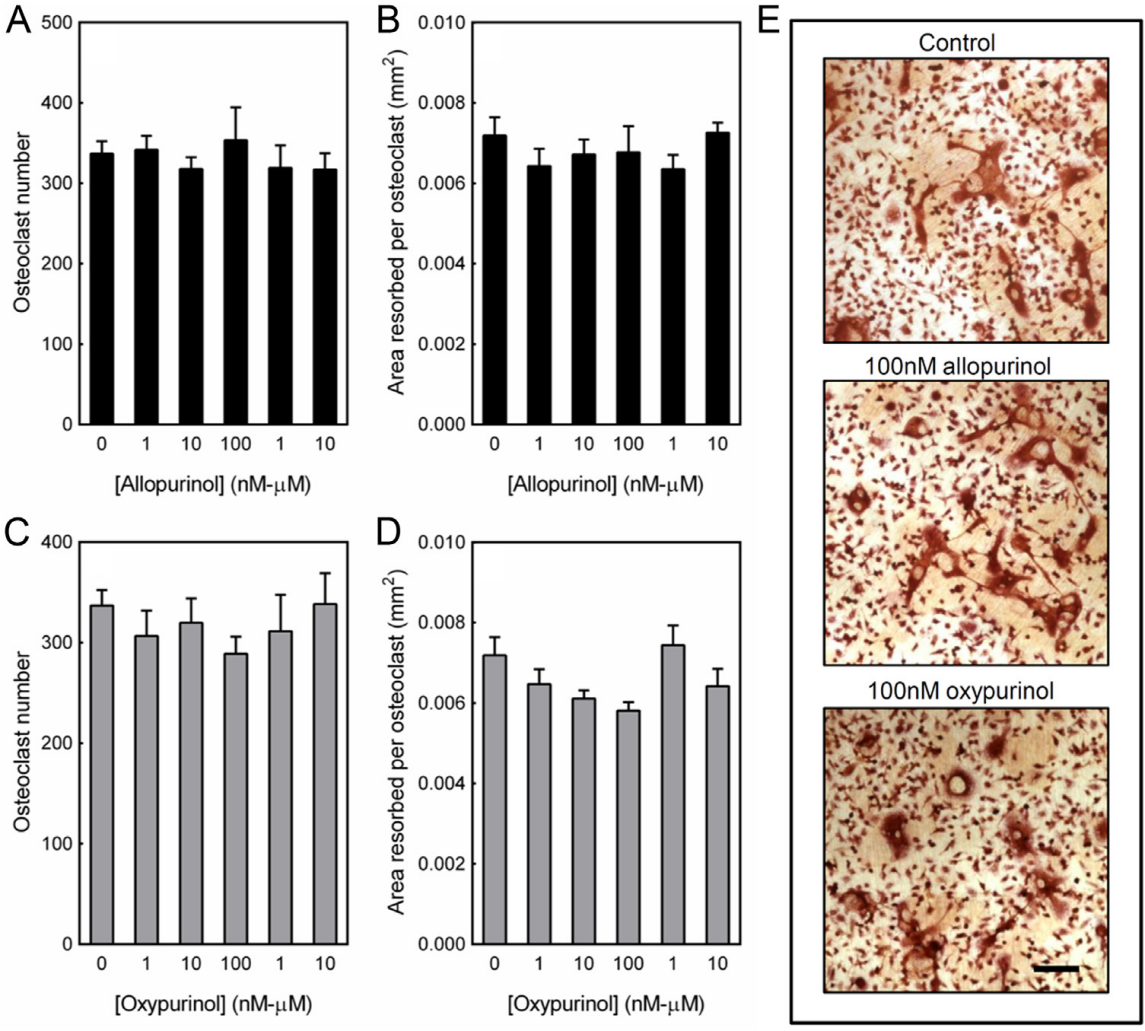
Osteoclast formation and activity is unaffected by allopurinol or oxypurinol. Mouse osteoclasts were cultured on dentine discs for 9 days with 1 nM–10 µM allopurinol or oxypurinol. At all concentrations tested allopurinol had no effect on (A) osteoclast formation and (B) resorptive activity. Treatment with oxypurinol also had no effect on (C) osteoclast number or (D) bone resorption. Values are means±SEM (n=8). (E) Representative light microscopy images showing no difference in osteoclast formation and resorptive activity. Scale bar=500 µm.

Mouse osteoclasts were treated with allopurinol or oxypurinol (1 nM–10 µM) for the duration of the culture. Both allopurinol and oxypurinol had no effect on osteoclast formation (Fig. 6A, C, E) or bone resorption (Fig. 6B, D, E).

## 4 Discussion

Allopurinol was first approved for use clinically in 1966 and is now a potential treatment for a range of conditions including chronic heart failure ischemia-reperfusion injury, vascular disease, chronic kidney disease and diabetes (see review by Pacher et al. [7]). Despite these additional uses, allopurinol remains one of the leading treatments for gout and hyperuricemia [4], [5]. Although gout is a condition that affects the musculoskeletal system, there is little published information about the direct actions of allopurinol and oxypurinol on bone cells. Our results show that allopurinol and oxypurinol increase osteoblast differentiation and bone formation *in vitro* but do not affect osteoclast function. In keeping with other studies showing that allopurinol and oxypurinol are well tolerated by cells and relatively non-toxic [24], [25], we observed no differences in osteoblast or osteoclast number with continuous treatment (≤10 µM).

Allopurinol and oxypurinol increased the expression of two markers of the mature osteoblast phenotype, TNAP and Ocn. TNAP activity was also increased. This suggests that a reduction in XO activity promotes osteoblast differentiation. The observation that XO knockout mice have reduced levels of adipose tissue and adipogenesis [26] is potentially consistent with this notion, since osteogenesis and adipogenesis are often inversely related [27], [28].

Available evidence suggests potential mechanisms by which allopurinol and oxypurinol may exert their osteogenic actions. Firstly, inhibition of XO leads to xanthine accumulation and a reduction in systemic uric acid levels [7]. Although the effects of uric acid on bone cell function have not been investigated, it has been reported that monosodium urate crystals, which form when uric acid exceeds its limit of solubility, are associated with decreased osteoblast viability and function [29], [30]. Secondly, XO inhibition by allopurinol and oxypurinol would also be expected to result in decreased production of hydrogen peroxide and O_2_^−^ free radicals. Previous work has shown that osteoblast differentiation and osteogenic gene expression are inhibited by these reactive oxygen species (ROS) [10], [11], [12]. Consistent with a role for ROS, the antioxidant vitamin E has also been shown to promote osteoblast function [31].

We also found that nanomolar concentrations of allopurinol, oxypurinol or febuxostat also markedly enhanced bone formation. This was due to an increase in both the number and size of the mineralised bone nodules formed. Since Col1α1 mRNA expression and production of soluble collagen were not significantly affected by allopurinol and oxypurinol treatment, our data suggest that the enhanced bone formation observed was not primarily due to increased organic matrix deposition. However, the increase in TNAP expression and activity combined with the decreased NPP1 expression suggest that allopurinol and oxypurinol could influence the level of bone mineralisation. NPP1 hydrolyses nucleotide triphosphates (such as ATP or UTP) to produce the key mineralisation inhibitor, pyrophosphate (PP_i_) [32], [33]; TNAP is the key enzyme involved in PP_i_ breakdown [34]. Thus, the opposing actions of NPP1 and TNAP are critical in determining the extracellular phosphate (P_i_)/PP_i_ ratio and, therefore, the level of skeletal mineralisation [34]. Taken together, our data suggest the stimulatory effects of allopurinol and oxypurinol on bone formation may be due to both increased osteoblast differentiation and a shift in P_i_/PP_i_ ratio in favour of bone mineralisation.

It has previously been reported that, despite being an effective drug, allopurinol is a relatively weak XO inhibitor *in vitro* (IC_50_ 0.2–50 µM) [7]. In agreement, we found that treatment with allopurinol or oxypurinol in the low micromolar range (0.1–1 µM) only reduced XO activity by up to 30%. However, at these concentrations both drugs increased bone formation by up to 4-fold. The apparent disparity between the level of enzyme inhibition and the actions of allopurinol and oxypurinol on osteoblasts suggests that some of the effects may be independent of XO inhibition. Febuxostat is a newer, more potent XO inhibitor which is structurally unrelated to allopurinol [6]. In comparative studies, we observed that febuxostat promoted bone formation to a greater extent than allopurinol, oxypurinol and a positive control for bone formation, BMP2. This observation provides additional support for the notion that the stimulatory effects are a consequence of XO inhibition; however, “off target” effects cannot be discounted.

The substrate for XO is xanthine, which is formed from the breakdown of extracellular ATP or adenosine. The key source of extracellular ATP in bone is controlled release from cells [35]. We found that ATP release was unaffected by allopurinol and oxypurinol treatment suggesting that inhibition of XO does not induce feedback mechanisms to prevent ATP efflux (and potentially xanthine accumulation). However, given that purinergic signalling is an important regulator of osteoblast differentiation and function [35], it is possible that blocking XO and the purine degradation pathway could indirectly influence ATP-mediated signalling and osteoblast activity.

In contrast to their potent actions on osteoblasts, we found that allopurinol and oxypurinol had no effect on osteoclast formation or resorptive activity. The mouse marrow cultures used here to study osteoclast function are relatively free of stromal cells and osteoblasts [21]. This suggests that under normal conditions, XO activity might not play a significant role in directly regulating osteoclast function. However, previous work has shown that XO-derived superoxide can stimulate RANKL expression in osteoblast-like cells [13]. Thus it is possible that XO could regulate osteoclast formation and activity indirectly via actions on other cell types. Furthermore, an earlier study found that allopurinol inhibits the increased bone resorption caused by TNFα and IL-1β [8]. Given that XO expression is significantly upregulated by stimuli such as inflammation and ischemia [36], [37] it is possible that the actions of XO on osteoclasts are only evident in inflammatory conditions.

Allopurinol is metabolised in the liver and has a half-life of 1–3 h in plasma, whilst oxypurinol is excreted in the urine and has a half-life of 12–17 h [38]. Typical plasma concentrations of oxypurinol are 30–100 µmol/L depending on the original dose of allopurinol or oxypurinol (100–400 mg) and the renal function of the patient [39]. The concentration of allopurinol or oxypurinol which bone cells are exposed to *in vivo* is unknown. However, since the skeleton is highly vascular and receives 7–8% of cardiac output it is possible that bone cells are exposed to the nanomolar/low micromolar concentrations tested in this study.

Allopurinol and oxypurinol exert their therapeutic actions by reducing plasma uric acid levels [7]. Clinical studies examining the relationship between serum uric acid levels (within the normal range) and BMD have yielded conflicting results. Several investigations report that higher serum uric acid levels are protective against osteoporosis [40], [41], [42], [43], [44], whilst others have found no effect [45]. In agreement with the suggestion that uric acid is protective against osteoporosis, Dennison et al. recently reported that high-dose allopurinol use in gout patients was associated with an increased fracture risk [46]. Furthermore, osteopenia has been associated with hereditary xanthinuria type II, which is characterised by defective XO activity and low serum uric acid levels [47]. In contrast, Basu et al. found that allopurinol use had no effect on hip fracture [48]. The results from these clinical studies are at variance to the striking osteogenic action of allopurinol and oxypurinol observed in this investigation. Our results also contrast with the *in vivo* study of Van den Bossche et al. [16], who found that the combination of allopurinol and another antioxidant, *N-*acetylcysteine, inhibited bone growth in an immobilisation-manipulation model of heterotopic ossification [16]. However, direct comparison between the two studies is difficult because of the large differences in methodology, dosing and the presence of a second antioxidant. Taken together, these data suggest that the role of XO in osteoblast differentiation and function is complex and the effects of XO inhibition on other cell types *in vivo* may exert additional actions that are not evident *in vitro.*

XO is widely expressed and its importance physiologically is highlighted by the XO knockout mouse model; these animals have very low serum uric acid levels and fail to thrive, typically dying before <9 weeks of age because of renal damage [49], [50]. XO knockout mice are also significantly smaller than wildtype animals [49], [50]. To date, there are no reports describing the effects of XO deletion on bone mass. Given the conflicting reports of the effects of uric acid on bone, skeletal analysis of these animals may provide further insight into the complex role of XO in bone under normal and pathological conditions.

## Conflict of interest

The authors declare that they have no conflict of interest.
